# Energetic Calculations to Decipher pH-Dependent Oligomerization and Domain Swapping of Proteins

**DOI:** 10.1371/journal.pone.0127716

**Published:** 2015-06-04

**Authors:** Prashant Shingate, Jim Warwicker, Ramanathan Sowdhamini

**Affiliations:** 1 National Centre for Biological Sciences, GKVK Campus, Bellary Road, Bangalore, 560065, India; 2 Faculty of Life Sciences, John Garside Building (MIB), 131 Princess Street, Manchester, M1 7DN, University of Manchester, Manchester, United Kingdom; Russian Academy of Sciences, Institute for Biological Instrumentation, RUSSIAN FEDERATION

## Abstract

Domain swapping mechanism is a specialised mode of oligomerization of proteins in which part of a protein is exchanged in a non-covalent manner between constituent subunits. This mechanism is highly affected by several physiological conditions. Here, we present a detailed analysis ofthe effect of pH on different regions of the domain swapped oligomer by considering examples which are known to be sensitive to pH in transiting from monomeric to domain-swapped dimeric form. The energetic calculations were performed using a specialized method which considers changes in pH and subsequent changes in the interactions between subunits. This analysis provides definitive hints about the pH-dependence switch from monomer to domain-swapped oligomer and the steps that may be involved in the swapping mechanism.

## Introduction

3D-domain swapping is an oligomerization mechanism in which two or more chains exchange their identical or similar structural elements. 3D-domain swapping term was coined by David Eisenberg [1]. Oligomerisation through domain swapping in some proteins depends on environmental factors like pH, temperature and mutation. pH is one of the crucial physiological factors which decide the fate of protein-protein oligomerization. There are natural pH gradients within the cell wherein different cellular compartments like endosomes differ in pH than that of cytoplasm and affect overall oligomerization process. In this study, we have examined energy considerations to observe pH-dependence of domain-swapping of proteins, whose structures are available in the Protein Data Bank (PDB) in both monomeric and domain-swapped form at widely different pH conditions. Few well-known proteins like RNAses [2–4], human cystatin C [3–6], Cyanovirin-N [7–13], which were experimentally shown to undergo domain-swapped oligomerisation by altering pH, were selected for the analysis.

Structure of RNase enzyme [14], the first domain-swapped molecule was available around five decades before, but still the swapping mechanism is not completely understood. This oligomerization process is either facilitated by formation of open conformers or by formation of more stable interactions or both. Such domain swapping mechanisms might be dependent on local environment such as pH. Alteration in pH causes changes in type and magnitude of interactions at the interacting regions. There is also a possibility that different interaction energy components contribute in various combinations with diverse magnitudes to facilitate domain swapping process.

Majority of the pH-dependent domain-swapped proteins undergo domain swapping at acidic pH. For instance, diphtheria toxin [1,14–16], exists as a stable but in active monomer at neutral pH and forms highly stable and active domain-swapped dimer once inside endosome at acidic pH after endocytosis. In the active domain-swapped dimeric form, the receptor-binding domains bind to the receptor to trigger a complex signalling cascade.

On the contrary, in few cases, proteins form stable domain-swapped structure at neutral pH and re-forms monomeric structures at acidic pH *e*.*g*. queen bee pheromone binding protein [17]. The pH-dependent domain swapping mechanism is utilized in many different ways in biological systems. In honeybee (*Apis mellifera*) colonies, the queen bee manages structure and activities of the hive by controlling the activities of the members. Queen bees secrete a major pheromone, 9-keto-2(E)-decenoic acid (9-ODA) which controls the worker bees and male bees, further modulating social or sexual responses. 9-ODA enters in the antennal lymph and binds to pheromone binding proteins (PBPs) whichtransport 9-ODA to the pheromone receptor in the sensory neuron membranes. The pheromone, once tightly bound to its PBP, is released to activate the receptor and this binding and release of pheromone is controlled by pH at different locations. At physiological pH,PBP viz. ASP1, exists as domain-swapped dimer and tightly binds to one or two 9-ODA molecules per monomer, while at acidic pH(in sensory neuronal membrane) dissociation of domain swapped dimer is coupled with release of 9-ODA. This pheromone then binds to pheromone receptor to initiate further signalling cascade.

**Fig 1:**
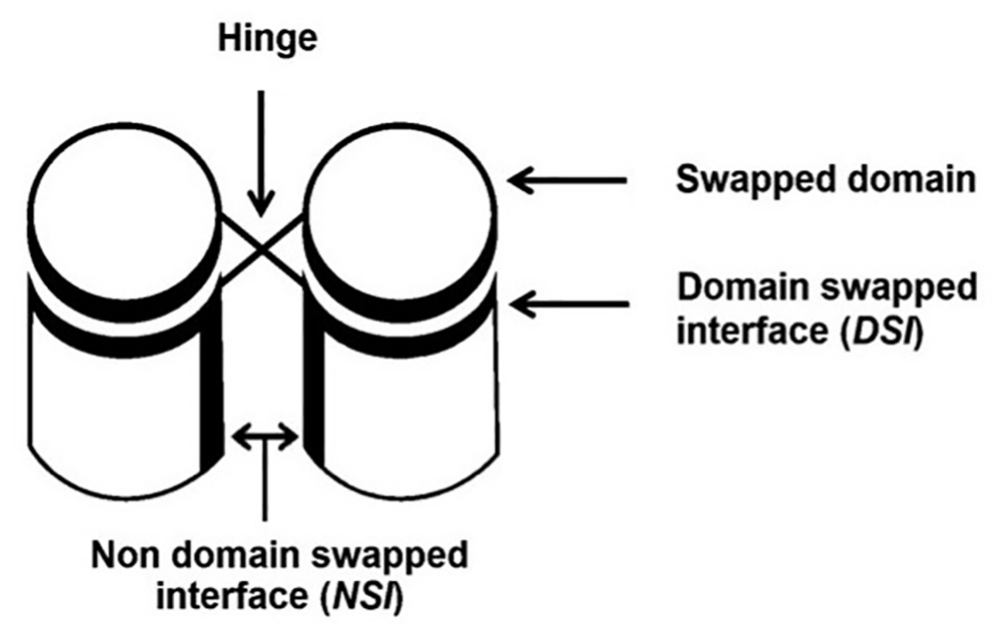
Domain-swapped oligomers with different regions marked.

3D-domain-swapped proteins contain two kinds of interfaces (Fig 1). One is domain-swapped interface (DSI) also called as closed interface [1, 17]. This interface is present in the monomeric form as intramolecular interface and retains in domain-swapped oligomeric form as intermolecular interface. Another type is non-domain swapped interface (NSI), which is a newly formed interface between two monomeric subunits in domain-swapped molecule due to proximity of monomeric subunits. DSI is present in both domain-swapped and non-domain-swapped form, hence its contribution to interaction energy may not be significant during domain swapping process [18,19]. Hence, newly formed interactions in NSI might be the key for the conversion of monomer to domain-swapped dimer.

We have examined the local energetics of domain-swapped dimer and non-domain swapped monomer at different interface regions for protein examples that are observed to exhibit pH-dependent domain swapping. pH-dependent energy calculation program [20,21] was employed for this purpose. This program was designed based on continuum electrostatic model. Continuum electrostatics models are highly helpful in analyzing pH-dependent properties: for instance, thermal free energy employed or released during different folding pathways at varying pH. When salt-bridges are considered individually, they are often weak and insignificant in folding or oligomerization process. However, their joint contribution is highly crucial in terms of maintaining thermal stabilities of folded protein subunit and protein-proteincomplex. For instance, several folded proteins lose their thermal stability at acidic or basic pH. This program has wide application to study neurodegenerative diseases associated with misfolding of proteins [5,8,22,23], pH-dependent enzyme activity, viral coat structure and intracellular trafficking.

Warwicker’s group has developed methods to calculate position and environment dependent pKa values and pH-dependent energy profiles for interacting entities. In this method, the pH-dependent pKa shifts were handled according to the extent of burial. This method has been developed by combining Finite Difference Poisson-Boltzmann and Debye-Huckel methods into a FD/DH algorithm. This method was successfully applied on various systems, for instance comparison of proteins of thermophilic and mesophilic organisms [24], binding of potassium ion in ion channels [25], and to study proteins according to their location in cellular compartments [26].

This pH-dependence program, which calculates pH-dependent energy profiles based on FD/DH hybrid scheme and also helpful in analyzing misfolded proteins, is highly useful to study pH-dependent domain swapping. As domain swapping process includes partial unfolding step prior to oligomerization, this method is highly relevant to this study. Therefore this program was applied on domain-swapped proteins and therespective non-domain-swapped forms available in PDB [27]. While selecting the dataset, we also ensured that both the monomeric and domain-swapped forms were crystallised under different pH conditions. The effect of pH change over wide range was observed on the thermal stability of DSI and NSI, by measuring the energetics at the interface in both domain-swapped and non-domain-swapped forms. Further, types of interactions stabilizing DSI or NSI were identified. The change in energy during formation of NSI and disruption of DSI appears to determine the rate of oligomerization through domain-swapping process.

## Materials and Methods

### Dataset

**Fig 2:**
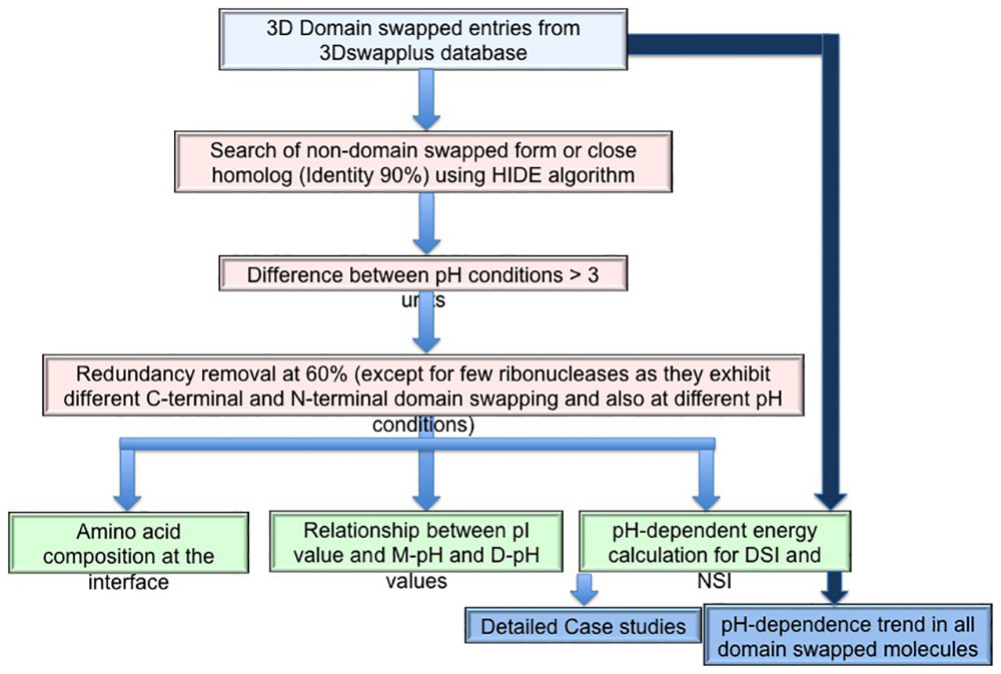
Strategy used to study pH-dependent domain swapping process.

All (2057) domain-swapped examples were selected from 3DSwapplus database [18,28]. Each entry was checked for availability of crystal structure of the same or close homologous protein (identity more than 90%) in non-swapped form, using an in-house HIDE server [18]. Non-domain-swapped forms could be identified for 732 domain-swapped protomers from this dataset. Further, only those pairs (of monomer-domain-swapped forms) which have a pH difference of 3 was considered and a redundancy filter (threshold sequence identity of 60% using Cd-hit program [29]) was applied to give rise to a final dataset comprising of 16 pairs (please see Fig 2 for a flow-chart of the steps involved).

A non-redundant dataset of domain-swapped proteins was obtained using entries from 3DSwapplus database to ensure same protein molecule is not repeated and the analysis is unbiased towards the limited protein structural availability. Redundancy check was performed on 2057 total entries from 3DSwapplus database, using Cd-hit tool at 60% threshold, leading to 1432 non-redundant domain-swapped entries. In the final dataset an exception was a RNase molecule which can form two distinct dimers under different physiological conditions involving domain swapping of two structural segments [30].

Datasets of general homodimers and transient oligomers, as employed by Srinivasan’s group [31], were used to compare pH-dependent energy profiles with that of the domain-swapped molecules.

### DSI and NSI

Boundaries of swapped domains were extracted from 3DSwapplus database. The interfaces in which no residue from swapped domain was involved were considered as NSI. New intermolecular interactions formed between swapped domains (here in only 1DDT) are considered as NSI. Residues within the two interfaces were identified using 7Å intermolecular distance criteria. Equivalence between residues from monomers and domain-swapped form were obtained using structural alignment. The percent composition for each type of amino acid was calculated for both and DSI and compared.

### Amino acid propensity calculations at DSI and NSI

Sequence of NSI and DSI interfaces were extracted from the dataset of pH-dependent domain swapped entries and non-redundant domain swapped in 3DSwapplus database. The amino acid propensity for each amino acid type was calculated using following statistical parameters:

AAPiPI=(niPIti)(NiPITi)

AAPiSI=(niSIti)(NiSITi)

$AAP_{i}^{PI}$ = Amino acid propensity of amino acid type “i” to be in NSI in pH-dependent domain swapped proteins with reference to general domain swapped proteins

$n_{i}^{PI}$ = Number of amino acid type “i” in NSI in dataset of pH-dependent domain swapped proteins

$N_{i}^{PI}$ = Number of amino acid type “i” in NSI in non-redundant dataset of general domain swapped proteins from 3DSwapplus database

$AAP_{i}^{SI}$ = Amino acid propensity of amino acid type “i” to be in DSI in pH-dependent domain swapped proteins with reference to general domain swapped proteins

$n_{i}^{SI}$ = Number of amino acid type “i” in DSI in dataset of pH-dependent domain swapped proteins

$N_{i}^{SI}$ = Number of amino acid type “i” in DSI in non-redundant dataset of general domain swapped proteins from 3DSwapplus database

*t*_*i*_ = Total number of amino acid type “i” in dataset of pH-dependent domain swapped proteins *T*_*i*_ = Total number of amino acid type “i” in non-redundant dataset of general domain swapped proteins from 3DSwapplus database

### Calculations of pH-dependent energy profile

**Fig 3:**
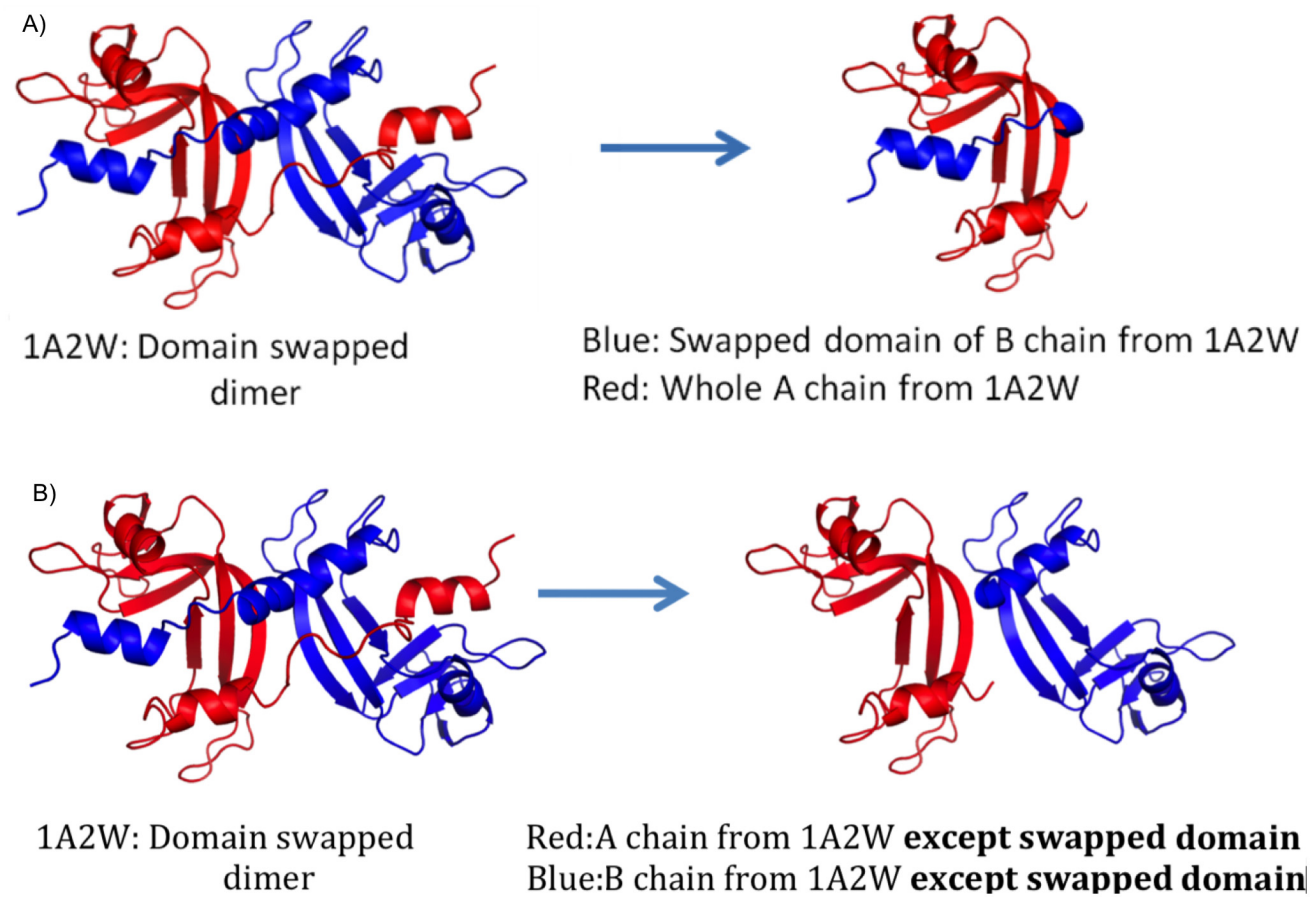
A) Input for pH-dependent energy profile for DSI using (Swapped domain N-terminal alpha-helix in this case). B) Input for pH-dependent energy profile for NSI (Excluding swapped domain N-terminal alpha-helix in this case).

pH—dependence program was applied for calculating and monitoring energies. This program requires interface between two protein structural entities as an input and employs Finite Difference method and Debye Hückel equation to calculate electrostatic interaction energy at varying pH [20,21]. This program also considers change in side chain conformation, change in ionization state of amino acids, varying dielectric constant according to position of the residue, etc. There is change in magnitude of interaction in response to the change in sidechain conformation. Most suitable rotamer for altered interaction is selected using mean field algorithm which considers solvent accessible surface area of polar and non-polar components of sidechains. Change in sidechain conformation causes changes in other interactions like Van der Waals interactions. Hence, all interactions will be calculated separately at different pH. pH-dependent energy profile of DSI for each domain-swapped oligomer in the dataset (swapped domain of one chain and its counterpart from another chain) were used as input (Fig 3A).

To estimate energy contributed by NSI at varying pH, protein complex without swapped domains from both monomeric subunits were used as input (Fig 3B). For diphtheria toxin alone (PDBID: 1DDT), we observed new interface formation (NSI) between swapped domains, hence pH-dependence profile for this NSI was also considered. Final output of the pH-dependence program provides energy profile of the DSI and NSI at varying pH.

An in-house server PPCheck [32], which calculates pseudo energy (a combination of electrostatic interaction, Van der Waals interaction and hydrogen bond energy) for each interface residue was further employed for detailed analysis.

## Results and Discussion

### Amino acid composition at interface regions

Analysis of amino acid propensities showed that DSI is rich in charged amino acids compared to general 3D-domain swapped proteins within pH-dependent domain swapped proteins (Table 1). This class of amino acids is the most sensitive to pH change. Before domain-swapped oligomer formation, DSI has to unfold. Hence, the higher presence of charged amino acids at this interface suggests that unfolding is majorly facilitated by pH change in DSI in proteins of the domain swapping kind. Besides, charged amino acids at DSI favoured few hydrophobic amino acids, suggesting weak hydrophobic interactions are preferred as they do not offer much hindrance during unfolding. These trends are not present for NSI.

**Table 1:** Amino acid propensity of DSI and NSI of pH dependent domain-swapped proteins with reference to non-redundant 3Dswapplus entries.

Residue type	Propensity in DSI	Residue type	Propensity in NSI
TRP	0.22	LEU	0.40
PRO	0.53	ARG	0.42
THR	0.54	PHE	0.43
LEU	0.59	ILE	0.57
GLN	0.62	VAL	0.64
CYS	0.84	TRP	0.70
ALA	0.89	GLN	0.72
PHE	0.97	ALA	0.74
SER	0.97	PRO	0.85
TYR	1.00	MET	0.99
GLY	1.11	GLU	1.07
VAL	1.15	TYR	1.19
MET	1.23	ASN	1.21
ILE	1.25	GLY	1.29
ARG	1.33	CYS	1.35
ASN	1.34	LYS	1.40
LYS	1.43	THR	1.40
GLU	1.43	ASP	2.14
ASP	1.93	SER	2.26
HIS	2.34	HIS	2.65

Both DSI and NSI interfaces favoured histidine. Histidine residue is well-known for its buffering action in response to pH change in biological system, as it possesses an imidazole nitrogen atom which has pK value close to physiological pH. For instance,in Semliki forest virus fusion protein, histidine residue was shown to be involved in regulating low-pH-dependent refolding[33]. Re-formation of DSI during domain swapping process is an important step and perhaps explains why histidine is observed in pH-dependent domain swapped proteins over general domain swapped proteins. Other polar amino acid residues are not as prevalent as histdine in both interfaces. In NSI, some amino acids like serine and threonine, which are prone to form strong hydrogen bonds in side chains, were favoured. Most of the hydrophobic amino acids were least favoured in both, as this class of amino acids is the least sensitive to pH change.

The order of amino acid types reported in Table 1 strongly agrees with the preferred order of amino acids according to point mutations at the interface in domain swapped protomers [7, 34–36]. Besides composition of the interface, the interactions between these interface residues are crucial as well. Hence, a change in behavior of these interactions according to change in pH were studied.

### pH-dependent energy profile of domain-swapped molecules

**Fig 4:**
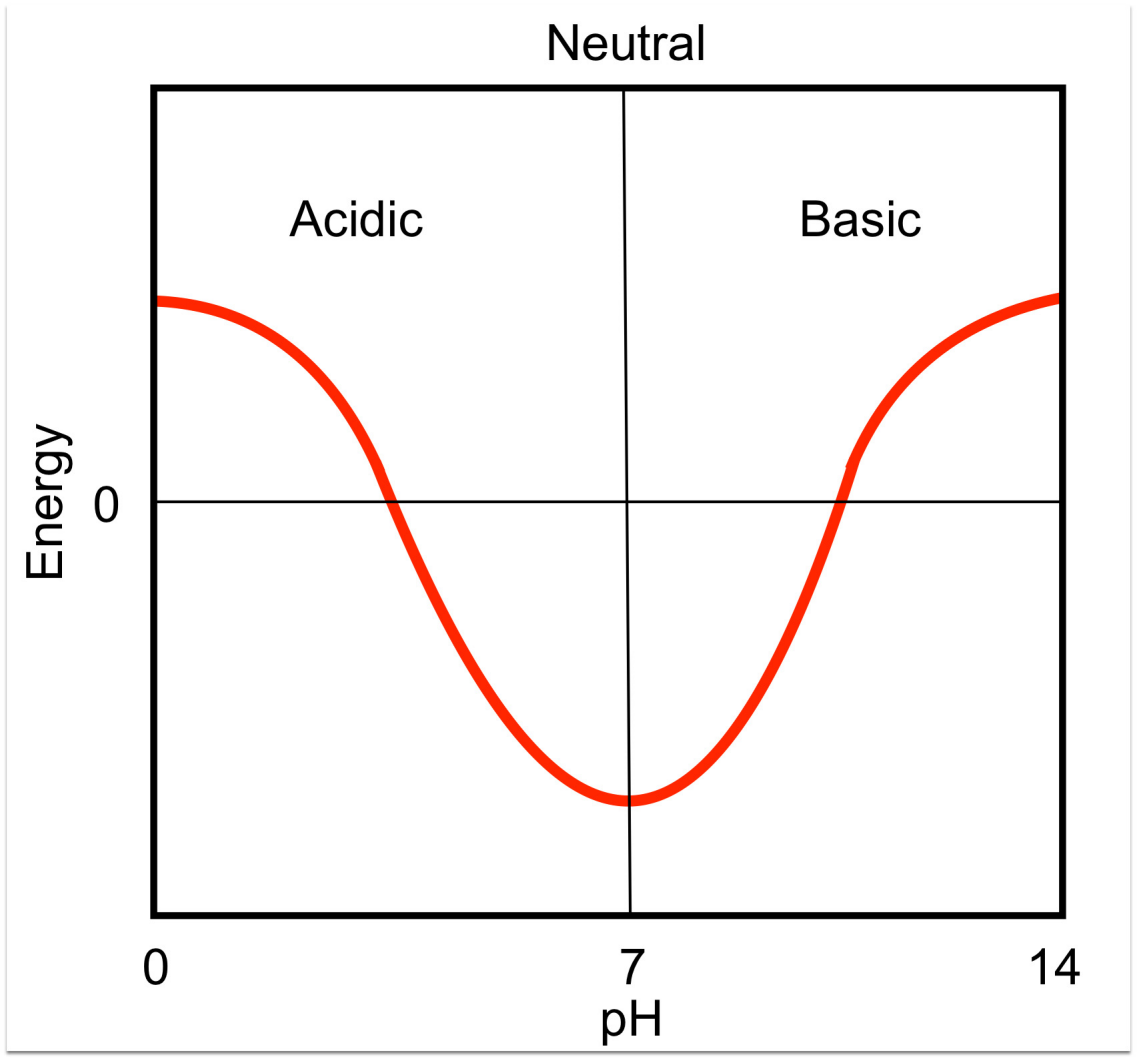
Classical pH-dependent profile.

pH—dependence program was used to calculate pH-dependent energy profiles for all 16 domain-swapped molecules (Table 2) that showed classical pH-dependence (Fig 4). pH—dependent energy profiles showed following trends at M-pH (pH at which given protein adopts monomeric form) and D-pH (pH at which given protein adopts domain-swapped form).

**Table 2:** Relative thermal stability of DSI and NSI at D-pH and M-pH.

Sr. No.	PDB ID	M-pH			D-pH		
		pH	Increase in thermal stability of DSI	Increase in thermal stability of NSI	pH	Increase in thermal stability* of DSI	Increase in thermal stability* of NSI
**Weakening of DSI and Strengthening of NSI**							
1	1DDT	7	Y	N	3.5	N	Y
2	1F0V	9.8	Y	N	6.5	N	Y
3	2XEW	7	Y	N	3.5	N	Y
4	3BCP	8.5	Y	N	5.5	N	Y
5	3DVH	3	Y	N	7	N	Y
6	3FKZ	5.5	Y	N	8.5	N	Y
7	3HAF	5	Y	N	10	N	Y
**Weakening of DSI only**							
8	1TIJ	8	Y	Y	4.8	N	N
9	3EK7	5.5	Y	Y	8.5	N	N
10	3GXY	6	Y	Y	10.3	N	N
11	1DZ3	7	Y	Y	4	N	N
12	11BA	8.4	Y	Y	4.8	N	N
**Weakening of NSI only**							
13	1A2W	4.5	N	N	7.5	Y	Y
14	3DVT	3	N	N	6	Y	Y
15	3E3I	3	N	N	7.5	Y	Y
**Negligible difference in energies at M-pH and D-pH**							
16	3DIE	8.5	N.A.	N.A.	4.6	N.A.	N.A.

*Thermal stability of interface means energy at that pH is lower than other pH (Thermal stability at D-pH means “energy of interface at D-pH” < “energy of interface at M-pH”).

In this study, 10 domain-swapped molecules showed strengthening of NSI, while 12 molecules showed weakening of DSI, at a pH where domain-swapped form is observed and structurally characterised (S1 File). Hence, it strongly suggests that weakening of interactions at DSI facilitates the formation of partially unfolded open conformers and increase in affinity within NSI at D-pH facilitates oligomer formation.

**Fig 5:**
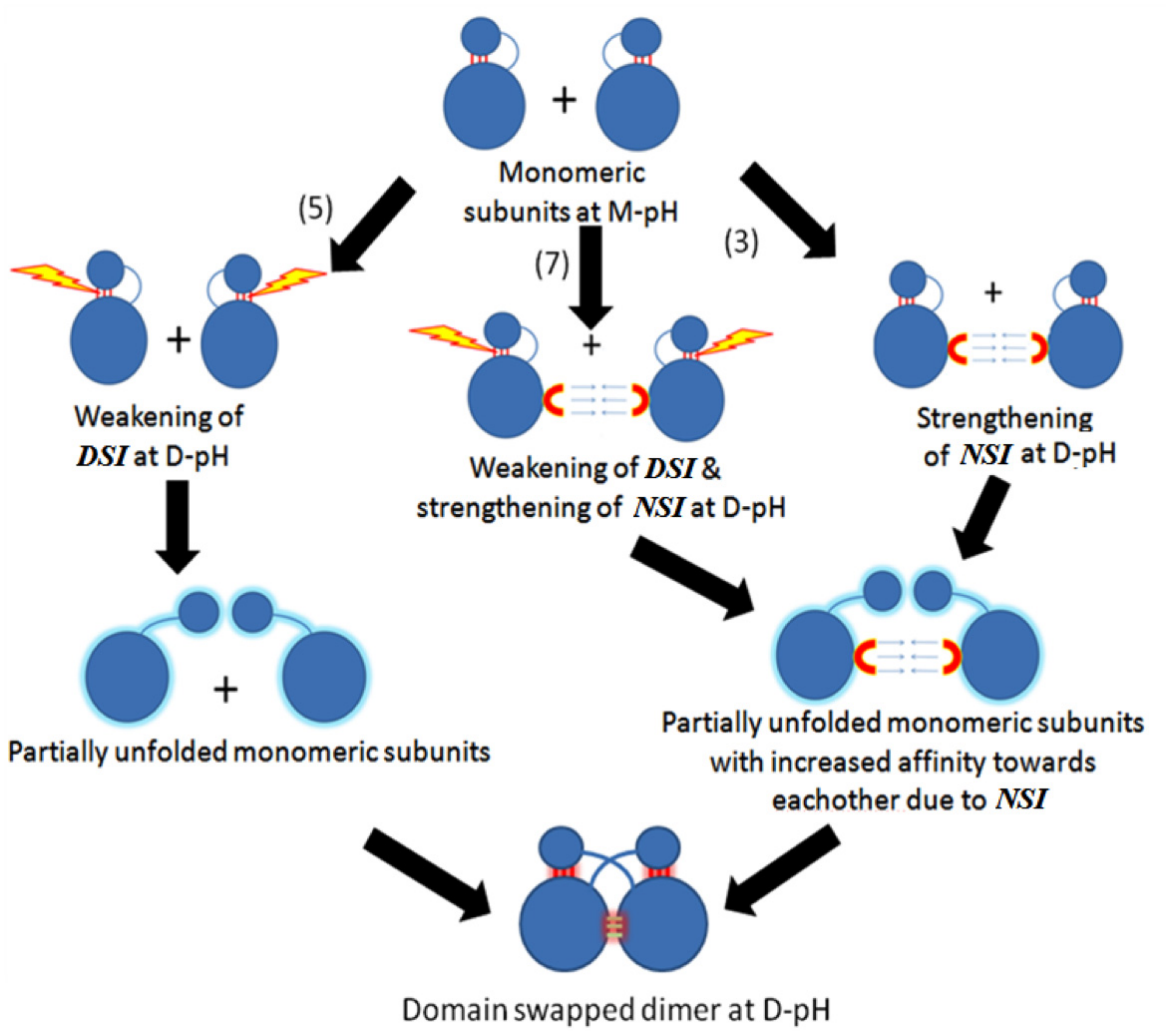
Proposed model representing possible effects of pH on DSI(SI) and NSI (PI) during pH-dependent domain-swapping.

Both weakening of DSI and strengthening of NSI were observed together in seven cases. Five cases showed only weakening of DSI, whereas strengthening of NSI alone was present in three cases (Table 2 and Fig 5). In only one molecule (3DIE), no significant change between energies at M-pH and D-pH for both the interfaces could be observed from our energy calculations.

Few structures were analysed in greater detail to obtain a structural rationale behind weakening of DSI and strengthening of NSI and how it might drive pH-dependent domain swapping.

### Case studies

#### 1) Diphtheria toxin

**Fig 6:**
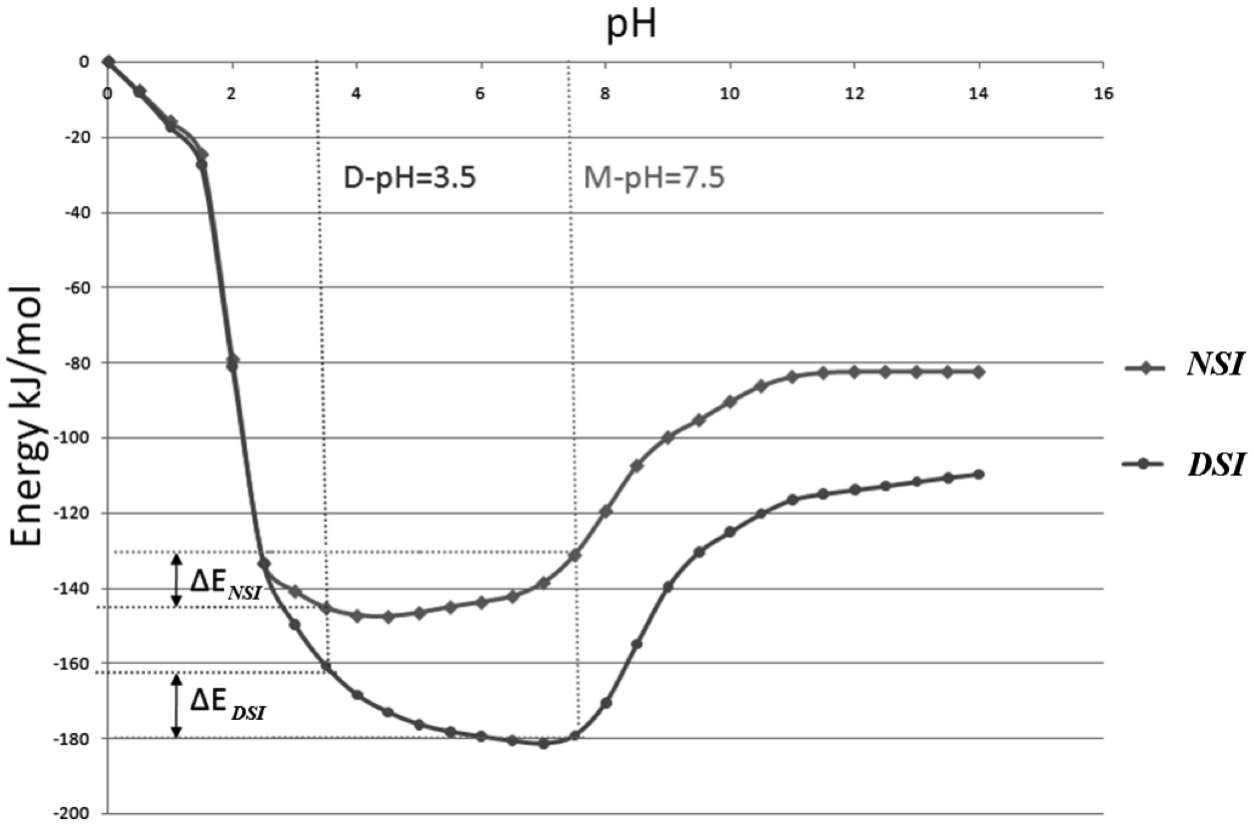
pH-dependent energy profile of diphtheria toxin (1DDT).

Diphtheria toxin (1DDT) is one of the best studied domain-swapped molecules. This protein undergoes domain swapping at acidic pH (3.6). Its pH-dependent energy profile showed both weakening of DSI and strengthening of NSI (Fig 6).

**Fig 7:**
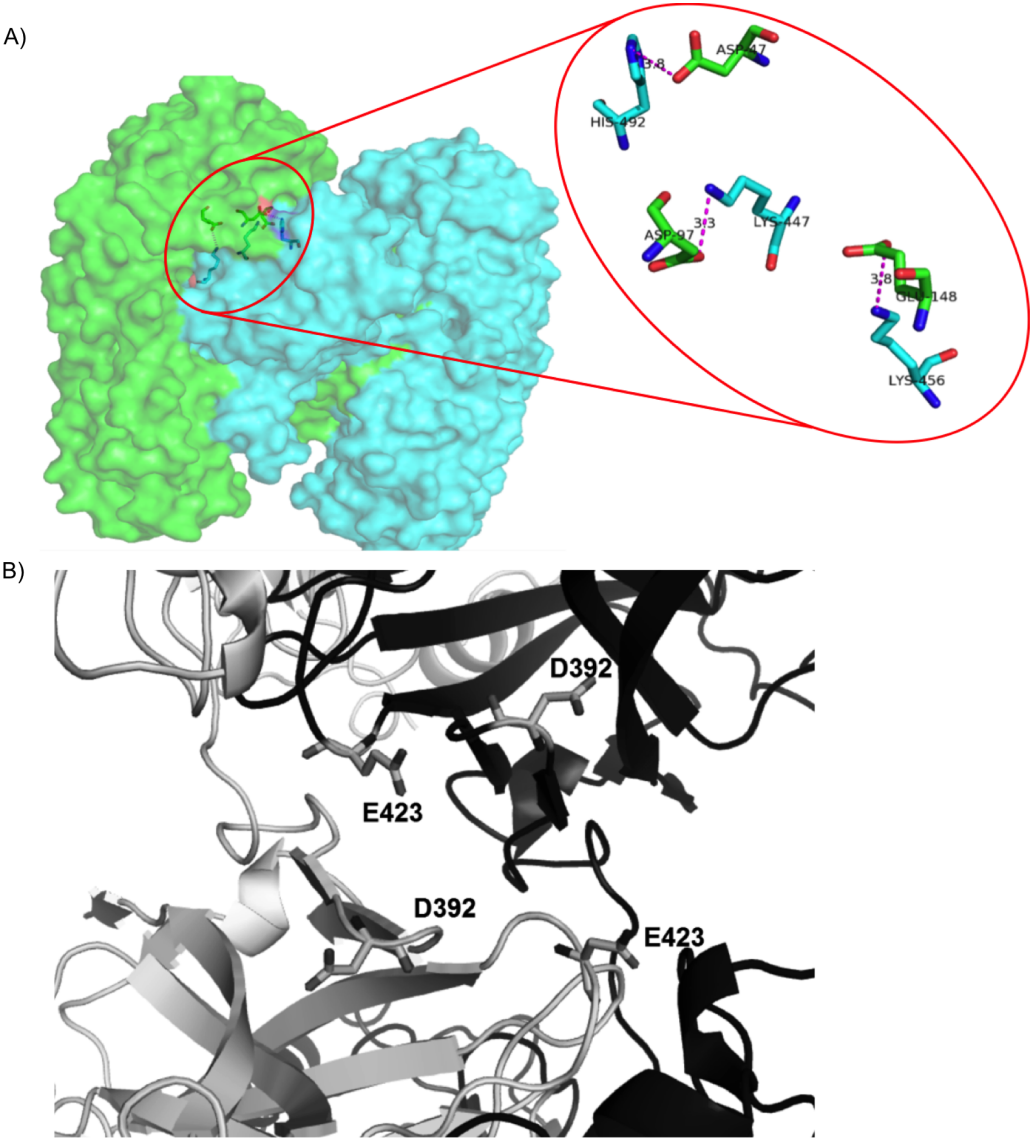
Interactions within domain swapped dimer of diphtheria toxin. A) Three salt bridges within DSI. B) Unfavourable electrostatic interactions between negatively charged residues within NSI.

A detailed structural analysis revealed that the major role for domain-swapping is played by negatively charged amino acids, Aspartic and Glutamic acid (Asp and Glu). Both Asp and Glu have isoelectric point near pH 3.6. Hence at D-pH, the net charge on these acidic amino acids is close to zero and all the salt bridges, where one of the interacting amino acids is either Asp or Glu, get severely weakened. There are three salt bridges present at DSI (Fig 7A). This is the major reason for weakening of DSI at D-pH.

While in pH-dependent energy profile of NSI, four negatively charged residues (Fig 7B) showed unfavourable interactions, when evaluated using in-house PPCheck[32] server. The magnitude of these unfavourable interactions drastically reduces at D-pH (around pH 3). Hence, reduction in electrostatic repulsion at NSI leads to strengthening of the NSI.

#### 2) Prion protein

**Fig 8:**
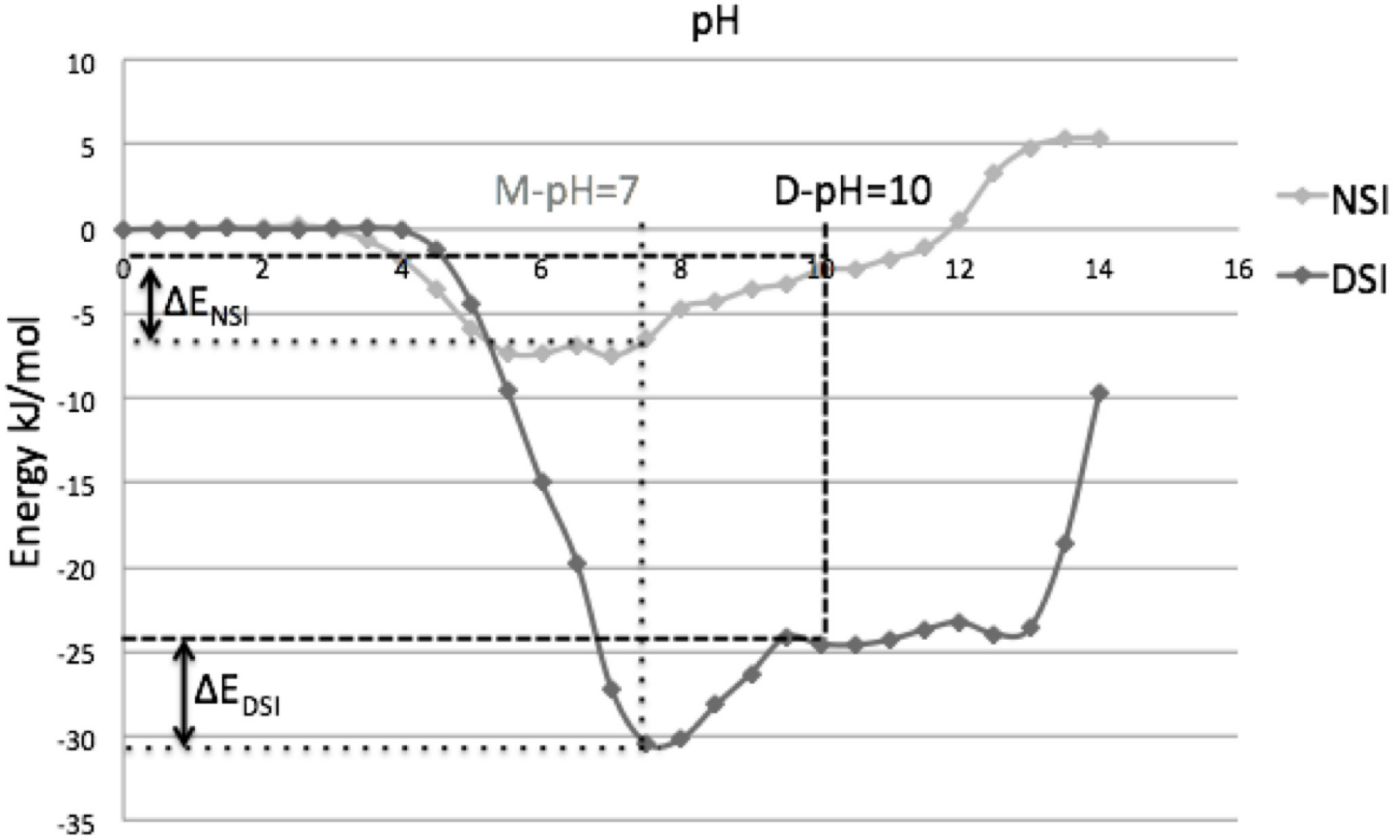
pH-dependent energy profile of prion dimer (3HAF).

Prion protein (3HAF) is one of the most important proteins due to its role in amyloid formation and neurodegenerative diseases. This protein forms domain-swapped dimer at basic (pH 10, referred as D-pH). The pH-dependent energy profile of this molecule showed both weakening of DSI and strengthening of NSI in D-pH (Fig 8).

**Fig 9:**
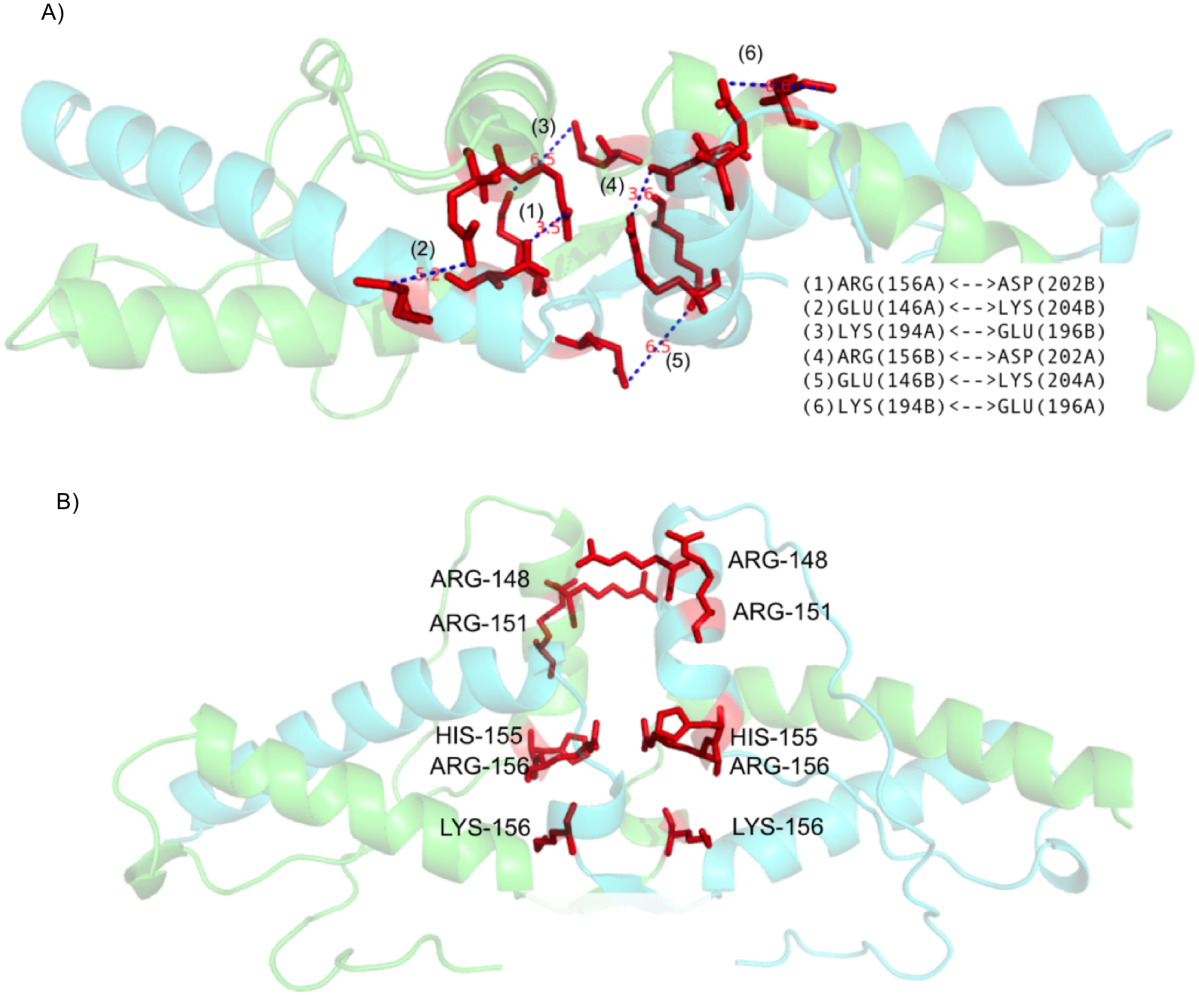
Interactions within domain swapped dimer of prion protein. A) Several strong and weak favourable ionic interactions between charged residues at DSI(shown in blue dotted lines). B) Several positively charged residues at NSI (shown in red color).

An in-depth structural analysis of the interactions in this protein dimer interface revealed that the crucial amino acids for domain-swapping are positively charged amino acids, predominantly arginine, moderately histidine and lysine (Arg, His and Lys) which provide salt bridge interaction at neutral pH. Both Arg and Lys have isoelectric point near pH 10. At D-pH, the net charge on these basic amino acids is close to zero leading to weak salt bridge interactions at D-pH (Fig 9A). pH-dependent energy profile of NSI and detailed analysis using PPCheck server [32] revealed unfavourable interactions between eight positively charged residues (Fig 9B). The magnitude of these unfavourable interactions drastically reduces at D-pH around 10, since net charge on most of these residues changed to zero leading to the strengthening of the NSI region.

In addition, two more pH-dependent domain swapped proteins (*viz*. ubiquitin and cyanovirin-N) showed similar behaviour as explained in cases above (please see S1 Text for details).

### Relation between pH-dependent change in DSI energy and oligomerization pH (D-pH)

**Fig 10:**
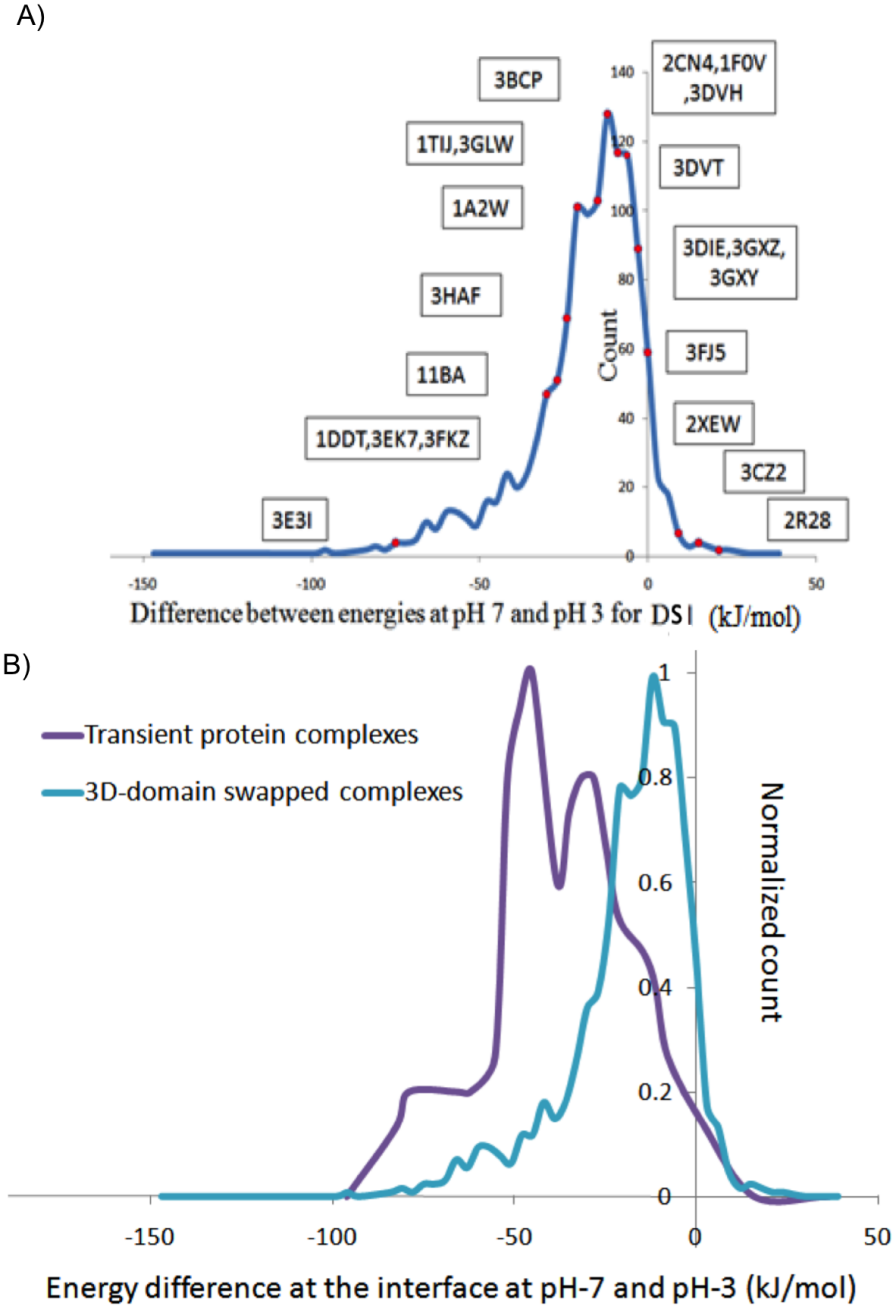
Distribution of difference between binding energies at pH 7 and 3. A) For DSI in 3Dswapplus entries. B) For DSI in 3DSwapplus entries and protein-protein interface in transient complexes

We observed that energy change from neutral to acidic pH (pH = 3) is a strong indicator of pH-dependence of a protein molecule. The plot (Fig 10A) represents distribution of energy difference of DSI of all domain-swapped molecules available. It revealed an interesting relationship between the change in energy and difference between experimentally observed domain-swapped pH and monomer pH, ΔpH. This plot showed very interesting trend after mapping very well-known literature-curated cases of pH dependence (shown in red circles in Fig 10A). This suggests a strong relationship between predicted energy difference at acidic and neutral pH with experimentally known pH of dimerization. DSI is present in monomeric as well as domain–swapped form. Hence, this pH-dependent energy difference has potential predictive application for inducing domain-swapping, through modulating pH conditions, if monomeric structure is available.

Since the occurrence of charged residues at the interface is observed in transient complexes as well, the distributions of energy difference of DSI at different pH of all domain-swapped molecules and interfaces from transient complexes were compared. In this analysis, we considered transient complexes since they associate to form a complex as well as dissociate into monomeric subunits at the same pH, unlike pH-dependent–domain swapped molecules. The dataset of transient complexes was chosen for this study as mentioned in Swapna and co-workers [31]. It was observed that their distributions were considerably different from each other (Fig 10B). Most of the transient dimeric molecules drastically lose their thermal stability, as pH changed to acidic pH compared to that of domain-swapped molecules. The reason might be the size of the interface, in most of the cases, DSI is formed by single secondary structural elements compared to whole interface of transient complexes. This observation suggests that it is very unlikely for monomeric subunits of transient complexes to oligomerize at acidic pH due to comparatively large reduction in thermal stability, while for 3D-domain swapped complexes, the difference in thermal stability is comparatively small at low pH and hence they are more likely to reform DSI.

pH-dependent change in energy also showed good correlation (correlation coefficient = 0.72) with number of salt bridges present at the DSI. Hence, the number of salt bridges present at DSI is another feature which can provide clues to predict D-pH for monomeric structure alone.

### Relation between pI of protein with D and M-pH

**Fig 11:**
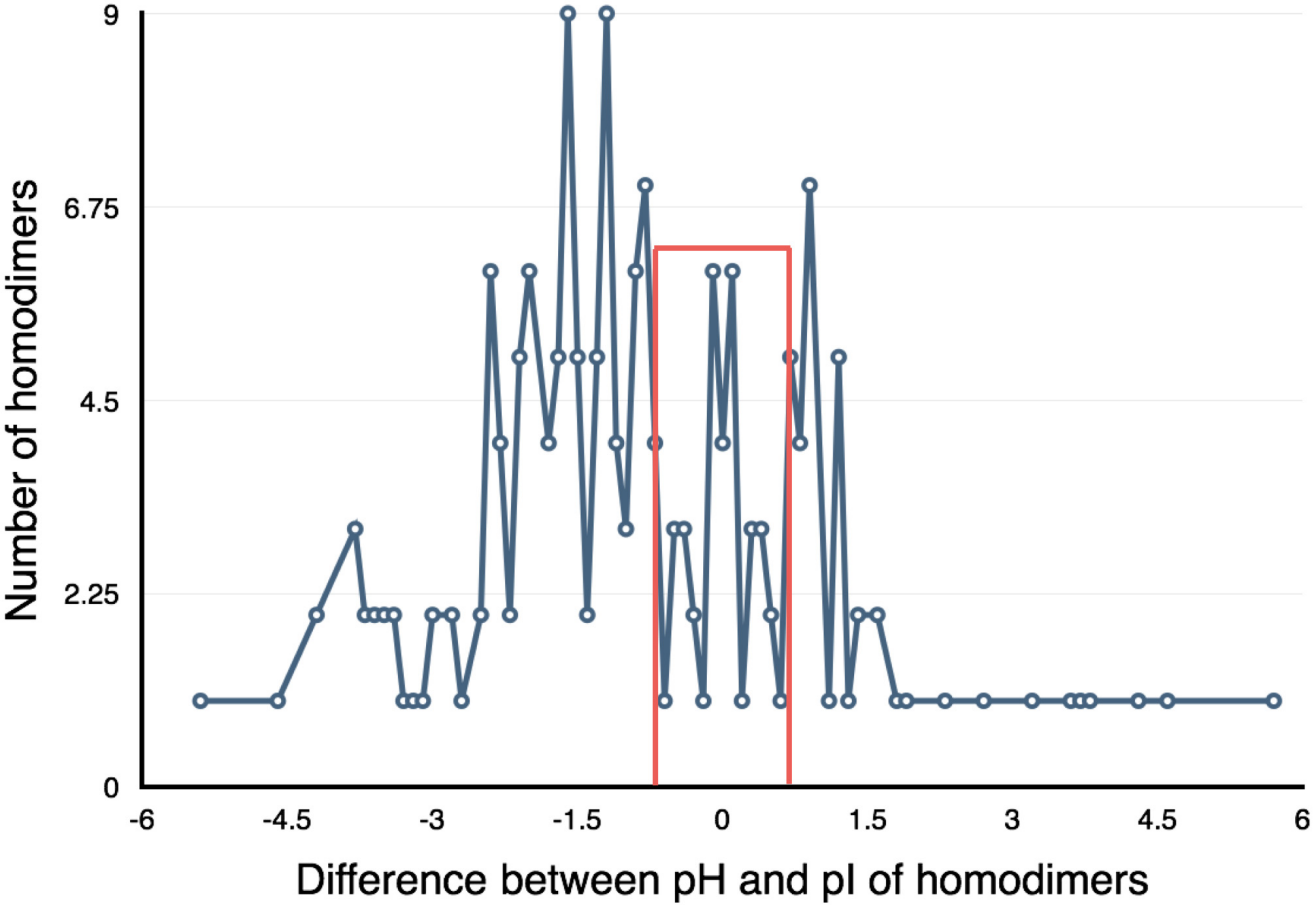
Distribution of difference between dimerization pH (D-pH) and pI in general homodimers.

The information of pH dependence for domain swapping might be hidden within protein sequence. Does pI of the protein indicative of pH-dependence of the protein? To check whether this characteristic is true for pH-dependent domain-swapped proteins or not, we further compared pI value of the protein and D-pH. It was observed that in eight proteins, out of 16 cases (50%), D-pH differs from to pI value by 1 (Fig 11, Table 3). While in three cases, it is close to M-pH and in five cases it is in between M-pH and D-pH. We also compared pI and D-pH value for general dataset of homodimers (as in [37]). In this dataset of general homodimers, on the other hand, only 9.5% have a pH difference less than 1 between pI and D-pH (please see Fig 11).

**Table 3:** pI, D-pH and M-pH values for pH-dependent domain-swapped proteins.

Sr. No.	PDBID	pI	M-pH	D-pH	Close to*
1	1DDT	5.93	7.5	3.5	-
2	3HAF	7.95	5	10	-
3	11BA	9.54	8.4	4.8	-
4	1TIJ	8.75	7	4.8	-
5	3GXY	4.94	6	10.3	-
6	3DVH	6.15	3	7	D
7	3E3I	6.3	3	7.5	D
8	1A2W	8.41	4.5	7.5	D
9	1F0V	7.34	9.8	6.5	D
10	1DZ3	4.98	7	4	D
11	3DIE	4.47	8.5	4.6	D
12	3DVT	6.26	3	6	D
13	3FKZ	8.35	5.5	8.5	D
14	2XEW	6.56	7	3.5	M
15	3BCP	9.04	8.5	5.5	M
16	3EK7	4.97	5.5	8.5	M

* D indicates that the difference between pI and D-pH is less than 1 while M indicates that the difference between pI and M-pH is less than 1

## Conclusions

We report a novel approach of using the tool of pH-dependence of interactions in our energy calculations to study different types of interfaces observed in few domain-swapped proteins. The amino acid propensity analysis suggests that charged residues and few polar residues at the interface are crucial for pH-dependent domain swapping. Abundance of these residues at DSI or NSI was a driving force for pH-sensitive domain swapping, while hydrophobic amino acids, least sensitive to pH change, are not favoured in the interface regions of pH-dependent domain swapped molecules. This analysis confirms that electrostatic interactions play an important role in pH-dependent domain swapping.

Further observation of these interactions at the interfaces revealed detailed insights of pH-dependent domain swapping process. It was observed that, in domain-swapped proteins, all protomers have to undergo partial unfolding by breaking of DSI in order to form domain-swapped molecule. Analysis of the pH-dependent energy profiles of such domain-swapped molecules showed weakening of DSI is one of the initial and essential steps in domain swapping. The energy required to break open or reform this DSI reduced after weakening of DSI at D-pH. Hence, despite negligible energy contribution in the domain-swapped complex, lowering the energy barrier required to break open DSI as a result of change in pH might be crucial event in pH-dependent domain-swapping. In majority of the cases under study, pH-dependent energy profile showed increase in thermal stability in NSI, this is another complementary feature to weakening of DSI which further increases in affinity between partially unfolded monomeric subunits towards each other and provides additional thermal stability to domain-swapped oligomers. In this study, we report the weakening of DSI and strengthening of NSI observed in majority of pH-dependent proteins. As these proteins were experimentally verified to be pH-dependent from earlier well-established studies, it further validates all the reported observations in this study. Amino acids at the interface were analysed in detail for few cases.

In 13 out of 16 cases in the dataset, domain-swapping was observed at acidic pH. However, it may not be possible to conclude that acidic conditions promote domain swapping in general. Further, pH-dependent energy profiles and pI values both were strong indicators of tendency of a protein to be engaged in pH-dependent domain swapping (D-pH). The analysis of behavior of DSI at different pH (especially at pH 3 and 7) showed a pattern in depicting D-pH. However, a detailed study is required for further understanding of this relationship and to develop a predictive tool if monomeric structure is available. Effects of pH on the hinge region can be explored to complement this study on interfaces and for availing structural insights towards the understanding of pH-dependent domain swapping.

## Supporting Information

S1 File pH-dependent energy profiles of all 16 pH dependent 3D domain swapped entries. (Figure A in S1 File) pH dependent energy profile of 1A2W, (Figure B in S1 File) pH dependent energy profile of 1DDT, (Figure C in S1 File) pH dependent energy profile of 1F0V, (Figure D in S1 File) pH dependent energy profile of 1TIJ, (Figure E in S1 File) pH dependent energy profile of 2XEW, (Figure F in S1 File) pH dependent energy profile of 3BCP, (Figure G in S1 File) pH dependent energy profile of 2CZ2, (Figure H in S1 File) pH dependent energy profile of 11BA, (Figure I in S1 File) pH dependent energy profile of 3DVH, (Figure J in S1 File) pH dependent energy profile of 3DVT, (Figure K in S1 File) pH dependent energy profile of 3E3I, (Figure L in S1 File) pH dependent energy profile of 3EK7, (Figure M in S1 File) pH dependent energy profile of3FKZ, (Figure N in S1 File) pH dependent energy profile of 3GXY, (Figure O in S1 File) pH dependent energy profile of 3HAF
(TIF)

S2 File Interactions within domain swapped dimer of Ubiquitin. (Figure A in S2 File) Two salt bridges within DSI in Ubiquitin. (Figure B in S2 File) Unfavourable electrostatic interactions between negatively charged residues within NSI in Ubiquitin.
(TIF)

S1 Fig Two salt bridges within DSI in Cyanovirin-N. (TIF)

S1 Text Additional case studies. (DOCX)
