# Supplementary figures and images for: Energetic Calculations to Decipher pH-Dependent Oligomerization and Domain Swapping of Proteins
Source: PLoS One. 2015 Jun 4;10(6):e0127716. doi: 10.1371/journal.pone.0127716 (PMC4456399; doi:10.1371/journal.pone.0127716)

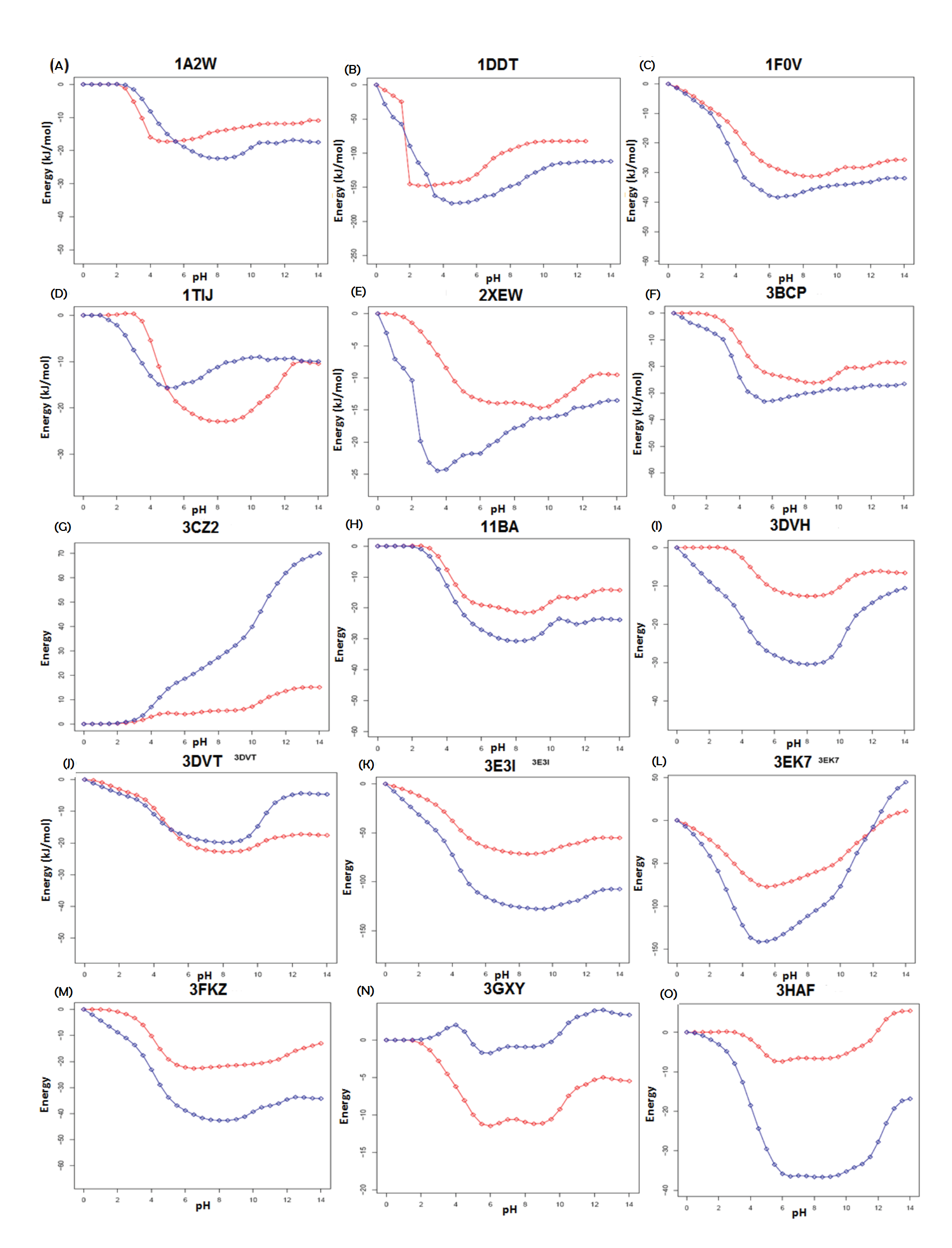

Supplement: S1 File — (Figure A in S1 File) pH dependent energy profile of 1A2W, (Figure B in S1 File) pH dependent energy profile of 1DDT, (Figure C in S1 File) pH dependent energy profile of 1F0V, (Figure D in S1 File) pH dependent energy profile of 1TIJ, (Figure E in S1 File) pH dependent energy profile of 2XEW, (Figure F in S1 File) pH dependent energy profile of 3BCP, (Figure G in S1 File) pH dependent energy profile of 2CZ2, (Figure H in S1 File) pH dependent energy profile of 11BA, (Figure I in S1 File) pH dependent energy profile of 3DVH, (Figure J in S1 File) pH dependent energy profile of 3DVT, (Figure K in S1 File) pH dependent energy profile of 3E3I, (Figure L in S1 File) pH dependent energy profile of 3EK7, (Figure M in S1 File) pH dependent energy profile of3FKZ, (Figure N in S1 File) pH dependent energy profile of 3GXY, (Figure O in S1 File) pH dependent energy profile of 3HAF (TIF) [file pone.0127716.s001.tif]

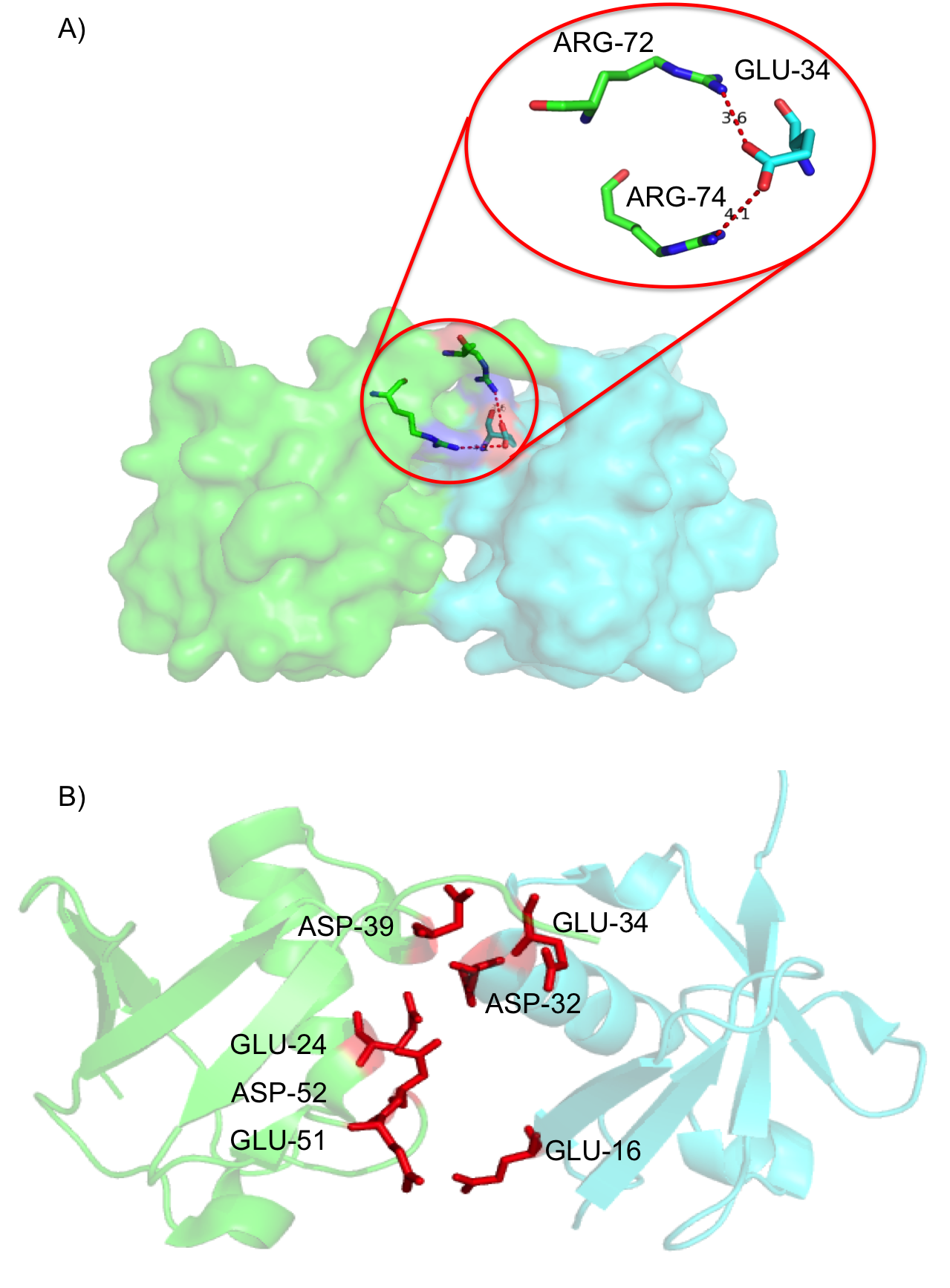

Supplement: S2 File — (Figure A in S2 File) Two salt bridges within DSI in Ubiquitin. (Figure B in S2 File) Unfavourable electrostatic interactions between negatively charged residues within NSI in Ubiquitin. (TIF) [file pone.0127716.s002.tif]

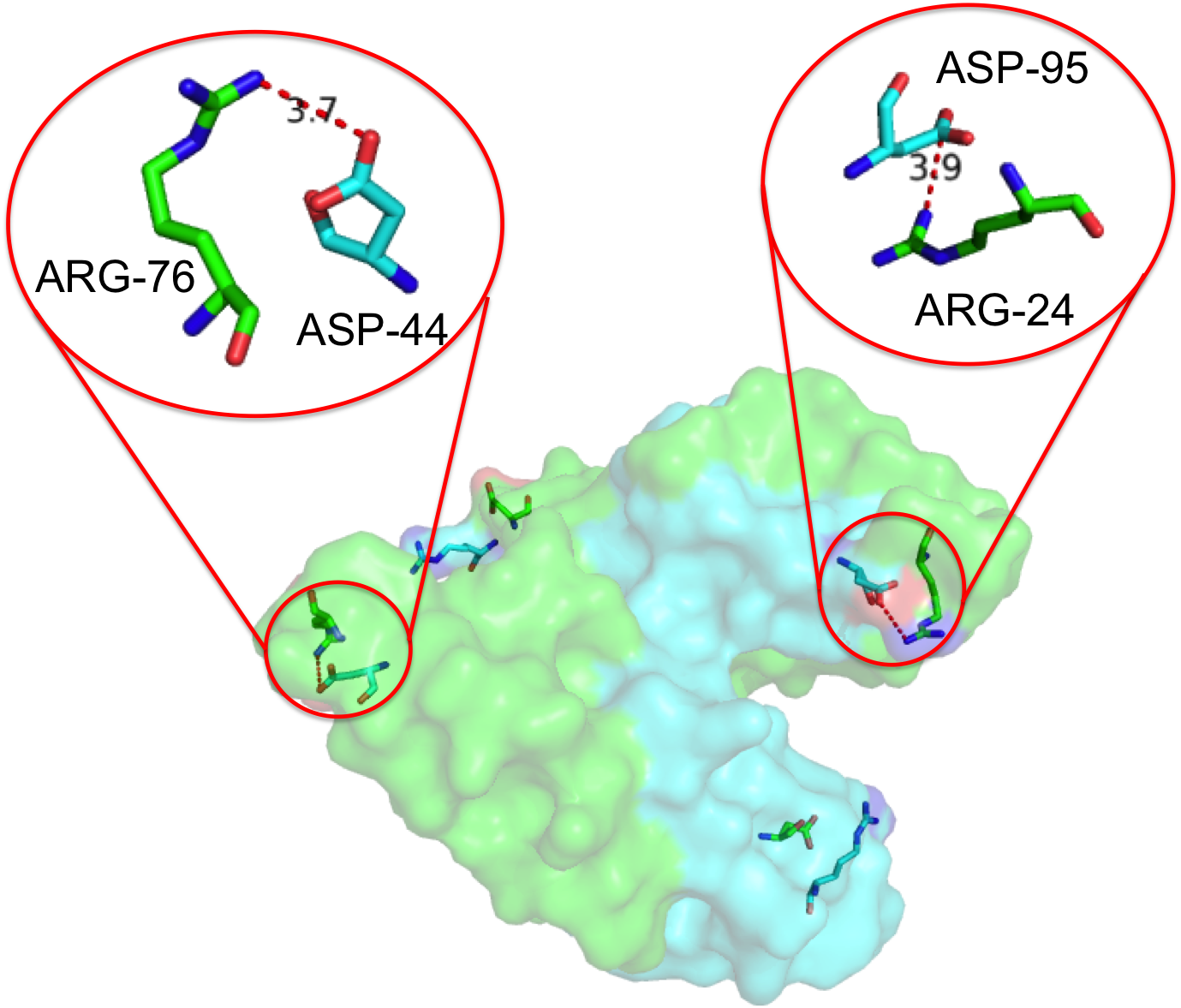

Supplement: S1 Fig — (TIF) [file pone.0127716.s003.tif]
