# Supplementary material for: Energetic Calculations to Decipher pH-Dependent Oligomerization and Domain Swapping of Proteins
Source: PLoS One. 2015 Jun 4;10(6):e0127716. doi: 10.1371/journal.pone.0127716 (PMC4456399; doi:10.1371/journal.pone.0127716)
Supplement: S1 Text — (DOCX) [file pone.0127716.s004.docx]

**Additional case studies**

**1) Ubiquitin domain swapped tetramer:**

Ubiquitin (2XEW) domain swapped form exists as tetramer. This protein undergoes domain swapping at acidic pH (3.5). Its pH-dependent energy profile showed both weakening of DSI and strengthening of NSI (Figure E in S1 File).

Similar to domain swapping process in diphtheria toxinit was observed that the domain-swapping is driven by negatively charged amino acids, Aspartic and Glutamic acid (Asp and Glu). Both Asp and Gluare acidic amino acids and have isoelectric point near pH 3.6. Hence at D-pH, the net charge on these acidic amino acids is approximately equal to zero hence all the salt bridges, where one of the interacting amino acids is either Asp or Glu, becomes weak. There are two salt bridges present at DSI (Figure A in S2 File). This is the major reason for weakening of DSI at D-pH.

NSI in ubiquitin tetramerpossesses seven negatively charged residues (Figure B in S2 File) and all together exhibitunfavourable interactions, when evaluated using in-house PPCheck [32] server. The strength of these unfavourableelectrostatic interactions decreases considerably at D-pH around 3.5. Hence, reduction in electrostatic repulsion within cluster of acidic amino acids at NSI leads to strengthening of the NSI.

**2) Cyanovirin-N domain swapped dimer**

Cyanovirin-N (3GXY) is one of the most important proteins as it’s a potent anti-HIV protein. This protein forms domain-swapped dimer at basic pH 10.5 (referred as D-pH). The pH-dependent energy profile of this molecule showed weakening of DSI (Figure N in S1 File).

An in-depth structural analysis of the interactions in this protein dimer interface revealed that the crucial amino acids for domain-swapping are two Arginine residues which provide salt bridge interaction at neutral pH.Arg has isoelectric point close to pH 10. At D-pH, the net charge on Arg is close to zero leading to weaker salt bridge interactions at D-pH (S1 Fig.). NSI of Cyanovirin-N hardly possess any charged amino acids hence lacks electrostatic interactions and hence is less affected by pH.
